# Small RNA Analyses of a *Ceratobasidium* Isolate Infected with Three Endornaviruses

**DOI:** 10.3390/v14102276

**Published:** 2022-10-17

**Authors:** Chi T. H. Cao, Mark C. Derbyshire, Roshan Regmi, Hua Li, Michael G. K. Jones, Stephen J. Wylie

**Affiliations:** 1 Plant Biotechnology Research Group—Virology, Western Australian State Agricultural Biotechnology Centre, Murdoch University, 90 South Street, Murdoch 6150, Australia; 2 Centre for Crop and Disease Management, Curtin University, Kent St, Bentley, 6102, Australia

**Keywords:** *Ceratobasidium*, *Endornavirus*, symbiosis, RNAi, small RNA

## Abstract

Isolates of three endornavirus species were identified co-infecting an unidentified species of *Ceratobasidium*, itself identified as a symbiont from within the roots of a wild plant of the terrestrial orchid *Pterostylis vittata* in Western Australia. Isogenic lines of the fungal isolate lacking all three mycoviruses were derived from the virus-infected isolate. To observe how presence of endornaviruses influenced gene expression in the fungal host, we sequenced fungus-derived small RNA species from the virus-infected and virus-free isogenic lines and compared them. The presence of mycoviruses influenced expression of small RNAs. Of the 3272 fungus-derived small RNA species identified, the expression of 9.1% (300 of 3272) of them were up-regulated, and 0.6% (18 of 3272) were down-regulated in the presence of the viruses. Fourteen novel micro-RNA-like RNAs (Cer-milRNAs) were predicted. Gene target prediction of the differentially expressed Cer-milRNAs was quite ambiguous; however, fungal genes involved in transcriptional regulation, catalysis, molecular binding, and metabolic activities such as gene expression, DNA metabolic processes and regulation activities were differentially expressed in the presence of the mycoviruses.

## 1. Introduction

Mycoviruses replicate intracellularly in fungi. They have been found in many pathogenic fungi, but few have been described from symbiotic fungi. Three mycoviruses classified in genus *Endornavirus* were described from a mycorrhizal fungus isolated from a wild *Pterostylis vittata* terrestrial orchid. The fungus was tentatively identified by ITS sequence as a species of *Ceratobasidium* [1]. Although Orchidaceae is one of the largest plant families (<30,000 species), and all members interact with mycorrhizal fungi for some or all of their lives, little research has been done to understand the roles of mycoviruses associated with orchid-fungus symbioses. 

Small RNAs are short non-coding RNA molecules 19 to 24 nucleotides (nt) in length. Two groups of small RNAs are dicer-dependent microRNAs (miRNAs) and short-interfering RNAs (siRNAs) [2,3,4]. miRNAs serve as regulators of endogenous genes while siRNAs are defenders of genome integrity in response to invasive nucleic acids such as viruses, transposons and transgenes. siRNAs were originally observed during transgene- and virus-induced silencing in plants (Mello and Conte, 2004). miRNAs are produced from an organism’s own genome from miRNA-coding genes. The short duplex RNAs are 21 to 24 nt, cleaved by RNase III-like endonucleases called dicers in animals and dicer-like proteins in plants and fungi, from their precursors, which are imperfectly base-paired hairpin structures for miRNA (Chen 2009) and fully complementary double-stranded RNAs for siRNAs [5]. Catalytic Agonaute proteins (AGO) help to remove one strand of the duplex RNAs resulting in mature miRNA/siRNA, which then associate with RNA-induced silencing complexes (RISCs). The miRNA/siRNA acts as a guide molecule to identify targets based on imperfect (in animals) or perfect/near-perfect (in plants) complementary base pairing to the sequences that are usually located at the 3′ untranslated regions (UTRs) of the target genes. This leads to translational repression and transcript degradation. 

Fungus-derived small RNAs, referred to as miRNA-like small RNAs (milRNA), were first identified in *Neurospora crassa* [6]. Since then, milRNAs and RNAi have been described in a number of fungal species [7,8,9,10,11,12,13,14]. The first example of RNAi as an antiviral defence mechanism was from the ascomycete *Cryphonectria parasitica*, the causal agent of chestnut blight [15]. This fungus used a subset of the RNAi machinery, dcl2 and agl20, to orchestrate an inducible antiviral defense response against the mycovirus Cryphonectria hypovirus 1 (CHV1) [15]. 

Recently, we used RNAseq to identify all the viruses infecting isolate C02 of an unnamed species of *Ceratobasidium*, an orchid mycorrhizal fungus isolated from a wild terrestrial orchid [1]. The three viruses identified were Ceratobasidium endornavirus B (CbEVB), Ceratobasidium endornavirus C (CbEVC), and Ceratobasidium endornavirus D (CbEVD), and they weakly suppressed growth of the host fungal mycelia under some laboratory conditions [16]. Little is known about the molecular interactions between endornaviruses and their hosts. We undertook a study to examine the influence of endornavirus infection on the expression of the fungal small RNAs in general and milRNA specifically. This information may provide insight into how mycoviruses manage to maintain their existence under targeted defense by their fungal host.

## 2. Materials and Methods

### 2.1. Fungal Isolate

*Ceratobasidium* isolate C02 was previously isolated from roots of a wild *Pterostylis vittata* orchid in 2012, and three endornaviruses were described coinfecting it [1]. Tentative identification of the fungus to genus *Ceratobasidium* was based on a 600 bp sequence of the internal transcribed spacer (ITS) region, using universal primers ITS1 (5’ TCCGTAGGTGAACCTGCGG 3’) and ITS4 (5’ TCCTCCGCTTATTGATATGC 3’). The fungal isolate was maintained in vitro on oatmeal agar at 4 °C. The presence of the viruses CbEVB (GenBank accession NC_031463), CbEVC (NC_031461), and CbEVD (NC_031449) was confirmed using RT-PCR with a species-specific primer pair targeting the *RdRP* gene of each virus (Table S1) [1]. 

### 2.2. Virus-Infected and Virus-Free Isogenic Fungal Lines

Development of an isogenic fungal culture lacking all three mycoviruses (designated ‘FREE’) derived from the virus-infected culture (C02) was described by us previously [16]. Three lines of each culture (virus-infected and virus-free) were chosen, and each of the six lines was tested regularly for the presence of each mycovirus, before and after treatments, using species-specific primers (as above). Virus-infected lines were named BCD2, BCD7, and BCD8 to designate that they were each co-infected with CbEVB, CbEVC and CbEVD. The three virus-free lines were designated FREE5, FREE6, and FREE8.

### 2.3. Fungal Genome Sequencing and Annotation 

*Ceratobasidium* isolate C02 was cultured in 25 mL potato dextrose broth (PDB) in a shaker at 100 rpm at 25 °C in the dark until 80–100 mg fungal biomass could be harvested. Genomic DNA was extracted using Trizol^TM^ (Invitrogen) by the method provided by the manufacturer. Genomic DNA (100 ng) was fragmented, libraries constructed and sequenced at the Australian Genome Research Facility. The genomic DNA library was constructed using the Truseq DNA nano library preparation kit (Illumina) following the manufacturer’s guidelines to create an average library insert size of 350 bp. The library was assessed by gel electrophoresis (Agilent D1000 Screen Tape Assay) and quantified by qPCR (KAPA Library Quantification Kits for Illumina). Paired-end sequencing of the library was performed on the NovaSeq 6000 S4 platform (Illumina) using 300 cycles chemistry. After sequencing, Truseq DNA sequencing adapters were removed, followed by quality trimming using default parameters within CLC genomics workbench (Qiagen). Trimmed reads shorter than 50 bp were removed. Overlapping reads were merged together to create longer contigs. De novo assembly was done in CLC genomics workbench using default parameters. 

### 2.4. Annotation of the Ceratobasidium Genome Assembly

Except where specified, all settings used with all programs were default. A custom library of repeats was created from the *Ceratobasidium* C02 genome using RepeatModeler version 2.0.1 [17]. This was combined with fungal repeats in the Dfam database [18] to create a unified database. RepeatMasker version 4.1.0 [19] was then used with the unified database to soft-mask the *Ceratobasidium* genome. Ab initio gene predictions were made on the soft-masked genome using a self-training hidden Markov model with GeneMark-ES version 4.59 [20].

All fungal proteins from SWISS-PROT [21] were then combined into a single FASTA file with amino acid sequences derived from coding sequence annotations of a selection of ten fungal genomes. These ten genomes were chosen based on the quality of their annotations and the fact that they were in Class Agaricomycetes class, to which *Ceratobasidium* belongs (Table S2). Proteins in the FASTA file were aligned to the *Ceratobasidium* assembly with a heuristic Smith-Waterman alignment using Exonerate version 2.4.0 [22], with the settings ‘--model p2g --showvulgar no --showalignment no --showquerygff no --showtargetgff no --targetchunkid 1 --targetchunktotal 100 --ryo “AveragePercentIdentity: %pi\n”’. These alignments were filtered using the Python script ‘filterExonerate.py’ (Supplementary File S1) to retain alignments with an average identity above 50%.

The filtered alignments made using Exonerate and the ab initio predictions made with GeneMark-ES were then used as input to the consensus gene caller Evidence Modeler version 1.1.1 [23], which was run on the soft-masked *Ceratobasidium* genome. To run Evidence Modeler, the different sources of evidence were weighted in the ‘weights file’ as follows: ab initio prediction = 1, protein alignment = 2. The consensus gene calls were converted to general feature format 3 (GFF3) format using the Evidence Modeler utility script ‘convert_EVM_outputs_to_GFF3.pl’ and their amino acid sequences were derived from this GFF3 in conjunction with the *Ceratobasidium* genome using the Evidence Modeler utility script ‘gff3_file_to_proteins.pl’. These amino acid sequences were then used as input to InterProScan version 5.48–83.0 [24], which was run with the settings ‘--formats TSV --goterms --iprlookup’ to ascribe functional domains and Gene Ontology terms. Concatenated coding DNA sequences were derived from the GFF3 annotations file and the *Ceratobasidium* genome using the Python script ‘fastaGff3ToCDS.py’ (Supplementary File S2). Gene annotation statistics were obtained using R package GenomicFeatures [25]. 

### 2.5. RNA Extraction and Small RNA Sequencing

Cultures were incubated in liquid glucose minimal media in the dark at 25 °C until 80–100 mg of fungal mycelium could be harvested. Total RNA was extracted using Trizol^TM^ Reagent (Invitrogen) by the method provided by the manufacturer, then treated with RNase-free DNase (New England BioLabs) to remove genomic DNA. The RNA was quantified by NanoDrop™ 2000 Spectrophotometer (Thermo Fisher Scientific, Waltham, MA, USA), and quality measured by a Agilent 2100 Bioanalyzer. For each sample, a small RNA sequencing library was prepared and enriched from 2 µg total RNA using the NEXTFLEX^®^ SRNA-seq kit v3 following the manufacture’s protocol. Total RNA was ligated to 3′ and 5′ adaptors. cDNA was synthesized from adaptor-ligated single-strand RNA, followed by amplification. Small RNA size-selection was done, aiming at a product band of 150 bp. After library preparation, six samples were pooled together, and single-end sequencing was done over 50 cycles on an Illumina HiSeq 2500 platform at the GENEWIZ Genomics Center (Shanghai, China).

### 2.6. Differentially Expressed Small RNA 

Bcl2fastq (v2.17.1.14) was used for base calling and preliminary quality analysis. The Fastq file for each small RNA sample was processed in CLC Genomics Workbench (Qiagen) to remove the NEXTFLEX^®^ 3’4N adenylated adapter using the adapter sequence: 5′ rApp /NNNNTGGAATTCTCGGGTGCCAAGG/3ddC/ followed by quality trimming using default settings. Reads of less than 18 nt and longer than 30 nt after adapter and quality trimming were removed. Annotating the *Ceratobasidium* genome was done with rRNA and tRNA based on the RFAM database with the program Infernal 1.1 [26]. All small RNAs that mapped to the Rfam database were removed from subsequent analysis.

The structured small RNA reads were then analyzed by ShortStack [27]. The RNAs of the six fungal lines (three with virus, three without), having lengths of 18 to 30 nt, were mapped to the *Ceratobasidium* genome assembly, allowing one mismatch per read to account for sequencing errors and single nucleotide polymorphisms (SNPs). Small RNA loci were identified. The threshold for the read coverage was 10 RPM. Reads in the 18–30 nt range were considered to be Dicer-derived reads. Strand cutoff was set at a default of 0.8, meaning a locus must have had 80% or more of its reads on the top strand to be called a positive-strand locus, or 20% or less on the top strand to be a negative-strand locus. All others receive no strand call (e.g., ‘.’). Only small RNAs with a strand call were eligible for miRNA prediction and analysis.

Read counts for all small RNA loci of the six replicate cultures were subjected to differential expression analysis using the R package DESeq2. Raw reads of each sample for each small RNA were normalized over the number of reads of each sample. Fold change (FC) of virus-infected samples over virus-free ones were calculated. *p*-values, attained by the Wald test were corrected for multiple tests using the Benjamini and Hochberg method to obtain adjusted *p*-values (Padj). Pdaj < 0.05 (or −log10(Pdaj) > 1.3) were considered statistically significant. A volcano plot displayed the differentially expressed sRNA loci. The plot shows statistical significance (−log10(padj)) on the y-axis against fold change of expression (log2(FC)) on the x-axis. SRNA loci were divided in to three groups: (1) up-expressed small RNAs: log2(FC) ≥ 0.6; (2) equally expressed small RNAs: −0.6 < log2(FC) < 0.6; (3) down-expressed small RNAs: log2(FC) ≤ −0.6. The up and down-expressed sRNAs have Padj < 0.05. 

### 2.7. Conserved miRNAs Screening and Novel milRNA Candidate Analysis 

To identify differentially expressed small RNAs, sequences of miRNA homologs, major small RNAs of differentially expressed small RNA loci, including over-expressed and under-expressed ones, were searched against miRBase (Release 22.1). Two mismatches or length differences were allowed for homolog determination. Novel miRNA-like small RNA (milRNAs) candidates were predicted by ShortStack using default parameters. ShortStack classifies small RNA loci into sixteen categories (identified as N1–N16), of which eight were identified from our *Ceratobasidium* small RNA data (Table 1).

Small RNAs in categories N14 and N15 were analysed for the secondary structure of their precursors using an online tool Mfold with default parameters [28]. The minimum free energy (MFE) of the hairpin structure was set as −20 kcal mol−1. GU is a wobble base pair with comparable thermodynamic stability to a Watson–Crick base pair. Thus, GU pairing was permitted in the Mfold criteria [14]. miRNA candidates, whose precursors had hairpin structures predicted by Mfold were considered to be novel *Ceratobasidium* candidate milRNAs. The expectation value was set at 3.5. The higher the expectation score, the less similarity between the small RNA and the target candidate. Thus, increasing the expectation value would increase undesirable random matches between the small RNA and the target. Therefore, we maintained the expectation value at 3.5. 

Target gene prediction was performed using psRNATarget online [29]. Differentially expressed milRNAs with statistical support (padj ≤ 0.05, either up-expressed with log2(FC) ≥ 0.6 or down-expressed with log2(FC) ≤ −0.6) were searched against the 31,294 coding sequences (CDS) and their annotations from the *Ceratobasidium* genome assembly we annotated as the target file. The maximum threshold of expectation value was 3.5 (above) and target accessibility-maximum energy to unpair the target site (UPE) threshold was set at 25; flanking length around target site for target accessibility analysis = 17 bp in upstream/13 bp in downstream and range of central mismatch leading to translational inhibition = 9–11 nt. Gene Ontology (GO) terms of the target regions, where they are available, were classified using the REVIGO web-based application [30].

## 3. Results

### 3.1. Ceratobasidium Genome Assembly and Annotation 

Small RNA profiles of the fungus were analysed under conditions of virus presence and virus absence. Due to the unavailability of a complete annotated genome and/or transcriptome of the fungal host, we sequenced the genome of Ceratobasidium isolate C02 [1]. 

The genomic DNA quality after extraction were assessed using a BioAnalyzer. DNA Integrity Number (DIN) was 7.1 out of 10, and the concentration was 85.3 ng µL^−1^. After the *Ceratobasidium* genome was shotgun-sequenced using the Illumina NovaSeq 6000 S4 platform, there was 28,005 Mb clean sequence (225× coverage), which was assembled into a draft genome of 93.3 Mb. The assembly was derived from 13,085 contigs of 1 kb or larger, with an N50 of 11.53 kb and a 50% GC content (Table 2). The *Ceratobasidium* C01 genome data is available at the NCBI database under BioProject PRJNA873516. The size of the *Ceratobasidium* C02 genome closely corresponded to the recently sequenced genome of *Ceratobasidium* sp. AG-Ba (96.29 Mb; GenBank accession: GCA_016906575.1), but larger than its close relative *Rhizoctonia solani*, which is 56 Mb (GCA_017311305.1). 

Gene prediction using a combination of the self-training hidden Markov model with GenMark-ES 4.49 and fungal protein alignments with the consensus annotation program ‘Evidence Modeler’ resulted in a prediction of 31,294 genes coding for 32,405 proteins. These amino acid sequences were used to search for homologous domains and functions from 13 public databases using InterPro. Of the 32,405 predicted protein sequences, 27,593 had at least one hit from a database. GO terms resulted for the amino acid sequences, where they are available, were classified using REVIGO, resulting in 751 GO terms related to biological processes, 290 to cellular components, and 893 to molecular functions. Proteins Argonaute (AGO), Dicer-like, and RNA-dependent RNA polymerases (RdRP) are major components in fungal RNAi machinery [31], thus it is important to know whether these proteins could be identified from this Ceratobasidium assembly. Eighteen amino acid sequences were found to be homologous to Argonaute binding/linker-like domains, three were homologous to Dicer-like domains, and thirteen contained RdRP-like sequences. Alignment of the AGO-like, Dicer-like and RdRP-like sequences of the Ceratobasidium C02 genome showed that they were non-identical.

### 3.2. Small RNA Analysis 

The assumption was that small RNAs of fungi are 18–30 nt in size [32,33]. Consequently, we selected a molecular size range of 18–30 nt for further analysis. To investigate the impacts of three endornaviruses on small RNA expression of the host, shotgun sequencing libraries of low molecular weight RNAs were created from total RNA isolated from mycelia of three virus-infected *Ceratobasidium* lines (BCD2, BCD7 and BC8) and three virus-free ones (FREE5, FREE6, and FREE8). The number of raw reads per sample was >12 million, the highest being BCD8 with a yield of 16.2 million (Table 3). After removal of low quality reads, reads shorter than 18 nt and longer than 30 nt, non-coding RNA and other structural RNA elements (reads mapped to Rfam databases, including tRNA, rRNA, snoRNA, snRNA, and siRNA), 30–40% of the total raw reads were retained (Table 3). Raw small RNA data of the six lines are available on the NCBI database under the BioProject PRJNA873516.

Small RNAs (Table 3) were aligned against the Ceratobasidium C02 genome assembly with one mismatch allowed. This analysis revealed a clear difference between the virus-infected (BCD) and virus-free (FREE) lines. The percentage of mapped reads in FREE lines was 95%, while 85% of clean reads in the BCD lines were mapped to the genome. 

**Figure 1:**
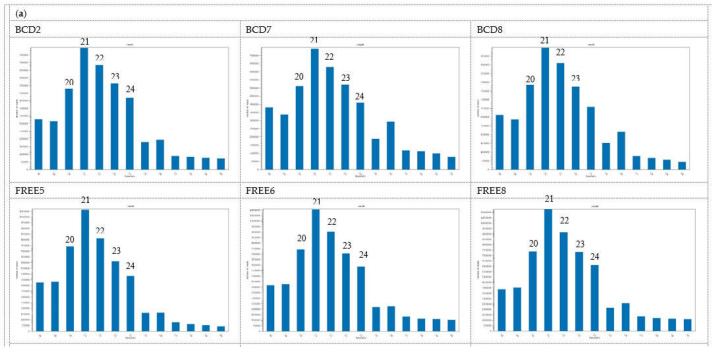

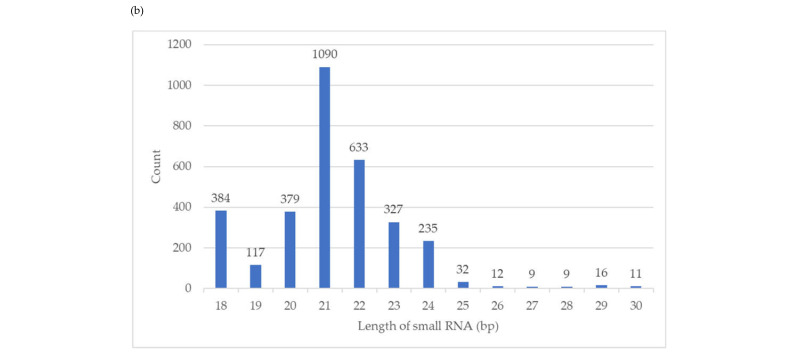
Size distribution of small RNA reads that mapped to the *Ceratobasidium* C02 genome assembly. (**a**) SRNA reads were trimmed of sequencing adapters, low quality bases, non-structure small RNAs removed, and reads of 18—30 nt selected for each of the six isogenic *Ceratobasidium* lines. Lines BCD2, BCD7 and BCD8 harboured three viruses, while lines FREE5, FREE6 and FREE8 were free of viruses (**b**) Size distribution of major small RNAs of 3272 small RNA loci, identified by ShortStack in the *Ceratobasidium* C02 genome assembly.

Length distribution of clean mappable reads that mapped to the Ceratobasidium genome assembly from each sample is represented in Figure 1a. The major proportion was in the 20–24 nt range, with 21 nt being most abundant in both BCD and FREE lines. There was an increase in the abundance of 26 nt sRNAs in the BCD lines, especially lines BCD7 and BCD8. 

Based on the ShortStack analysis, 20,092 putative small RNA loci were identified. Of those, 3272 loci had coverage larger than or equal to 10 RPM. Among these, 248 loci were derived from the sense strand, 279 were from the antisense strand, and 2745 loci were strand-undetermined. The length of loci ranged from 20 nt to 65.5 kb. Major small RNAs for each locus and the single most abundant RNAs were identified. The length distribution of the major small RNAs (Figure 1b) was consistent with the Ceratobasidium mapped reads (Figure 1a), with 21 nt being the most abundant, followed by 22 nt.

### 3.3. Global Differential Expression of Small RNAs

**Figure 2:**
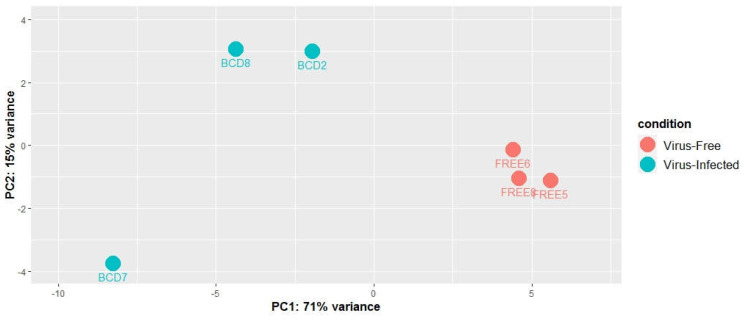
Principal components analysis (PCA) model built on the normalized counts of 3272 small RNA loci of three virus-infected fungal cultures (BCD) and three virus-free fungal cultures (FREE).

The differential expression of small RNAs between BCD and FREE lines were analyzed. Counts of the major small RNAs from the 3272 small RNA loci were used for normalization, then differential expression analysis was done using the R package DESeq2. Principal component analysis (PCA) was done using normalized counts of the major small RNAs to assess the differences between the virus-infected samples and virus-free samples and to identify the most differentially expressed small RNA loci (Figure 2.). The PCA plot clearly showed the two groups creating two distinct clusters, indicating that small RNA profiles of virus-infected and uninfected cultures differed. The x axis describes 71% of the culture variance, showing that small RNA expression variation between virus-infected and virus-free cultures is a major contributor to the overall variance. However, there also seems to be a considerable amount of variance explained by differences between virus-infected cultures. Compared to the virus-infected ones, the small RNA profile of culture BCD7 differs from the other two BCD cultures. These differences are also apparent on principal component 2, which explains about 15% of the variance.

**Figure 3:**
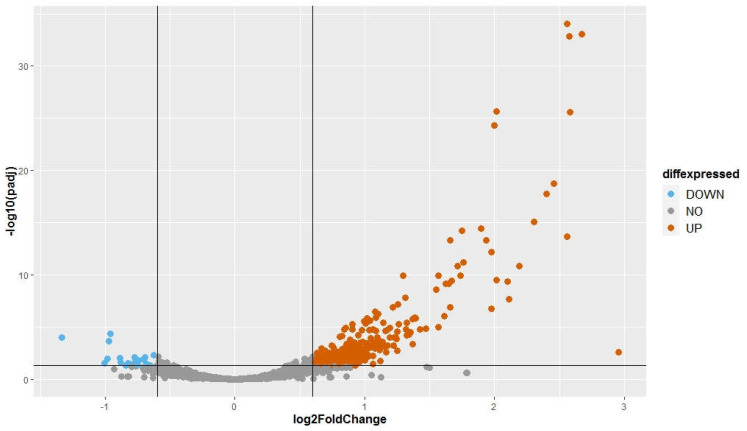
Volcano plot presenting the differential expression analysis of 3272 small RNA loci with a coverage ≥ 10 rpm. Fold change was obtained by comparing normalized reads counts of three virus-infected samples over the three virus-free samples. The adjusted *p*-values attained by the Wald test were corrected for multiple testing using the Benjamini Hochberg method (DeSeq2 package). A locus was considered to be statistically differentially expressed when its log2 (FC) ≥ 0.6 or ≤−0.6 and -log10 (padj) ≥ 1.3.

The fold change (FC) of normalized expression of BCD samples to normalized expression of FREE samples was calculated for each major small RNA of the 3272 small RNA loci. We used a FC cut off of 1.5 (log2(FC) = ±0.6). Adjusted *p*-values (padj) were used with a threshold at 0.05 to identify statistically significant differences in expression. Small RNAs were classified into three groups: (1) upwardly expressed small RNAs: log2(FC) ≥ 0.6; (2) equally expressed small RNAs: −0.6 < log2(FC) < 0.6; (3) downwardly expressed small RNAs: log2(FC) ≤ −0.6. Among the small RNAs analyzed, there were 300 upwardly expressed small RNAs, 2954 equally expressed small RNAs, and 18 downwardly expressed small RNAs (Figure 3). The small RNA expression differences between two groups of samples suggest that the infection by three endornaviruses changes how Ceratobasidium expresses its small RNAs. The differentially expressed small RNAs are given in Table S3.

### 3.4. Differentially Expressed miRNA-like Small RNAs 

Identification of conserved miRNAs among 318 differentially expressed small RNA, including 300 (9.1%) upwardly regulated and 18 (0.6%) downwardly regulated small RNAs was done by searching the small RNAs against miRBASE (release 22.1). No homologs were found. 

Differentially expressed small RNAs were analysed by ShortStack to identify novel miRNA-like small RNAs (milRNAs). This resulted in no milRNA candidates which passed all the tests, including sequencing the exact miRNA-star. However, as milRNA the prediction function in ShortStack is built to analyse plant miRNAs, it could miss fungal miRNA-like candidates. We decided to undertake a further analysis of mature miRNAs as predicted by ShortStack. ShortStack miRNA prediction classified the miRNA candidates into sixteen categories, and five of them are possible miRNA candidates (N11 to N15). Among 300 up-regulated small RNAs, 91 of these were predicted as possible milRNAs, falling into N11 to N15 categories. No down-regulated small RNAs were predicted (Table 4). The small RNA loci in categories N11 to N15 have low complexity (Table 5), which indicates loci are dominated by just a few RNAs. This increases the likelihood that these loci are true small RNA loci.

**Figure 4:**
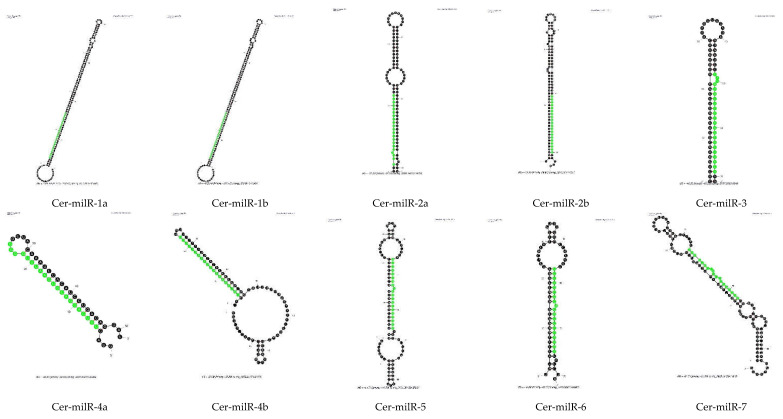

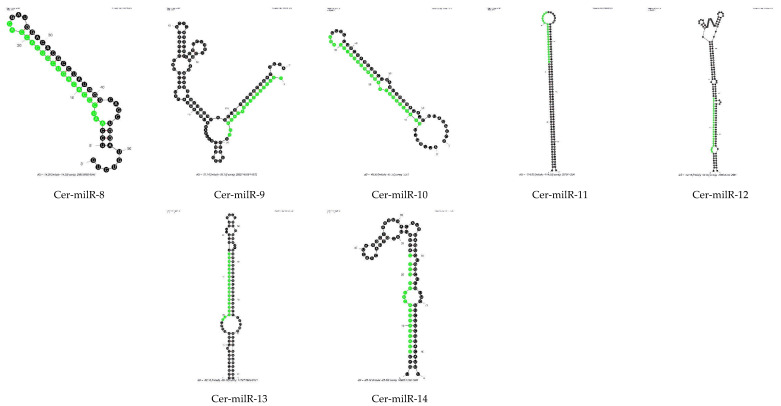
Prediction of hairpin structures of *Ceratobasidium* C02 pre-milRNAs. The secondary structures were processed with the Mfold application with default parameters. The sequences of possible milRNAs are highlighted in green.

One of the characteristics of miRNAs is their precursor molecules form a hairpin structure [32,33]. We used Mfold to predict secondary structures. Two important criteria were used: the length of the precursor and the minimal free energy (ΔG). As the length of pre-milRNAs in N. crassa was about 38–160 nt [34], it suggests that in fungi, the length of pre-milRNAs could be quite long, so we set the lengths of the whole hairpin and hairpin loop to not exceed 300 nt. According to ShortStack analysis, N11, N12 and N13 milRNA candidates did not meet the requirements regarding the secondary structure of their precursor molecules; hence, we did not process the RNA folding assessment for these milRNA candidates. Only the precursors of N14 and N15 milRNA candidates were assessed for hairpin structure by Mfold using default parameters. Thirteen small RNAs were classified in N14 and N15 categories by ShortStack. The Mfold models showing the secondary structures of thirteen N14 and N15 milRNA candidates is presented in Figure 4. They all show putative hairpin structures of the milRNA precursors. 

After prediction of the secondary structure of N14 and N15 putative milRNAs, we named the novel miRNA-like candidates of Ceratobasidium C02 as Cer-milRNAs (“Cer” for Ceratobasidium), which is consistent with the nomenclature used in N. crassa [14,34] and in Trichoderma reesei [14] (Table 5). The Ceratobasidium milRNA sequences were used to search miRBase [35] to find homologues. There were no homologous sequences of miRNAs identified in other organisms, including filamentous fungi.

The novel Cer-milRNAs mainly originated from the minus strand of non-coding regions, apart from Cer-milRNA-5 from contig 3301. The Cer-milRNA-5 precursor, located in the region from nt 38,139–38,234 of contig 3301 was locating on a coding sequence with function annotation predicted by InterPro as an aspartic peptidase A1 domain (InterPro ID: IPR021109). Among these seven milRNAs candidates, Cer-milR-1, Cer-milR-2, and Cer-milR-4, each originates from two different milRNA precursors. Half the Cer-milRNAs had a 5′ uracil. 

Of fourteen novel milRNA of Ceratobasidium C02, seven Cer-milRNAs were up-regulated (UP) upon infection by the viruses, especially Cer-milR-4, which was expressed four-fold higher (log2FC = 2) in the virus-infected samples (Table 5). Cer-milR-1, Cer-milR-2 and Cer-milR-4 from both precursor locations were significantly up-regulated. 

### 3.5. In Silico Target Gene Prediction of Differentially Expressed Ceratobasididum milRNAs

Prediction of target genes may provide insights into the fungal host’s biological functions and pathways where the milRNA is functioning. As there is no Ceratobasidium cDNA library available on psRNATarget, we used the list of 31,294 final coding sequences (CDSs) and their annotations from the Ceratobasidium genome assembly we annotated as the target file. We were only interested in differentially expressed Cer-milRNAs (seven of them) and their potential target genes as these could give insights into which biological functions and pathways of the fungal host three endornavirus have impacts on.

A list of the predicted targets of the seven up-regulated Cer-milRNAs and their biological functions is given (Table S4) with the threshold expectation = 3.5 and UPE = 25, we found that six of them bind to at least one target (Cer-milRNA-1: 5 genes, Cer-milRNA-2: 9 genes, Cer-milRNA-3: 11 genes, Cer-milRNA-4: 5 genes, Cer-milRNA-5: 3 genes, Cer-milR-6: 1 gene). Interestingly, we could not find possible targets of Cer-milR-7 with these above parameters. 

For functional classification of the target genes, GO terms of the predicted target genes of Cer-milRNAs (where they were available) were analysed by the online tool REVIGO [30] (Table S5). Importantly, the targets included transcription factors, Leucine-rich repeat (LRR) containing proteins like protein kinase and F-box proteins. We also found that Cer-milRNA-1 and Cer-milRNA -3 target to Enhancer/Suppressor mutator (En/Spm)-like transposable elements and the Tetratricopeptide-like helical domain superfamily (Table S5). Function classification of GO terms shows they are mainly related to molecular functions such as transcription regulator activity, catalytic activity and molecular binding activity, and biological processes such as metabolism and gene expression.

## 4. Discussion 

### 4.1. Evidence of RNAi Machinery in Ceratobasidium sp.

RNAi is a highly conserved defense mechanism against invading RNA elements found in most, if not all eukaryotic organisms, including fungi. In this study, we found evidence of the RNAi machinery in an orchid mycorrhizal fungus of genus Ceratobasidium. The annotated genomic assembly of Ceratobasidium contained homologous sequences of the three essential RNAi proteins, Dicer, Agonaute and RdRP. Eighteen Ceratobasidium amino acid sequences were homologous to the Argonaute binding/linker domain, three were homologous to Dicer domains, and thirteen contained RdRP-like sequences. These key components of the RNAi machinery are not universally present in all fungi, including Saccharomyces cerevisiae [36]. Recent analyses of fungal genomes of some pathogenic fungi show evidence of RNAi machinery. In Fusarium graminearum, genome analysis identified two Dicer proteins (FgDicer1 and FgDicer2), two Argonaute proteins (FgAgo1 and FgAgo2), and five RNA-dependent RNA polymerases (FgRdRP1–5) [12]. Five Argonautes, two Dicers, and four RdRPs were predicted to be encoded by the genome of F. oxysporum [36], and two Dicer-like and three Argonaute-like proteins were present in the Trichoderma reesei genome [14]. Bioinformatic analysis predicted that Sclerotinia sclerotiorum genome encodes two Dicer-like proteins and at least one Argonaute protein. These proteins play a crucial role in RNAi [13,37]. A Penicillium chrysogenum genome analysis revealed two Dicer-like proteins [38]. The number of AGO-like proteins and RdRP-like proteins found in Ceratobasidium in the present study may be higher than for other fungi, but only three Dicer-like proteins were identified. If the RNAi machinery in Ceratobasidium processes in the same manner as in other organisms, in which each Dicer is responsible for the generation of one miRNA type, this suggests that three milRNA types exist in Ceratobasidium.

Comparison of Cer-milRNAs against miRBase revealed no homologs of conserved miRNAs of plants, animals, and other filamentous fungi. Because of the evolutionary divergence between miRNAs, there is no common miRNA between plants and animals despite similar mechanisms of miRNA generation [39]. This result is consistent with some other fungi, such as F. oxysporum [11].

Previous studies indicate that the loading of a small RNA onto an AGO protein is guided by the 5′ end nucleotide of the small RNA. Some AGO proteins show 5′ end nucleotide preference, which is typically uracil [40]. In the ascomycete N. crassa, Argonaute-like proteins interact with a class of small RNAs called qiRNAs, with a 5′-terminal uracil [41]. AGO2 and AGO4 of Arabidopsis thaliana predominantly favor small RNAs starting with the 5′-terminal A, AGO1 preferentially recruits small RNAs initiating with a 5′-terminal U, while AGO5 predominantly binds small RNAs with a 5′ -terminal C [40,42]. It is still unknown which of the remaining AGOs (if any) could preferentially bind small RNAs with a 5′ -terminal G. The selectivity of AGO proteins is different in Drosophila, where AGO1 recruits small RNAs beginning with U, whereas small RNAs binding to AGO2 most frequently begin with C [43]. 

In our study, half the Cer-milRNAs identified had a 5′ uracil, one of the signature indicators of true miRNAs in other organisms, including Penicillium chrysogenum [38]. This indicates that AGO guided by 5′ uracil in Ceratobasidium could be the dominant one. Other Cer-milRNAs ending at 5′ with other nucleotides could be loaded onto other AGO proteins. 

The small RNA population of Ceratobasidium was similar in length distribution to those of plants, namely 20–24 nt [44]. Studies of other fungi revealed a predominance in the range of 19–22 nt in F. oxysporum [11], T. reesei [14], P. marneffei [45] and S. sclerotiorum [13]. However, the lengths of predicted Cer-milRNAs was quite variable, from 18–24 nt, which is not the typical size distributions of miRNAs in plants (21–24 nt) [44] or animals (22 nt) [4].

### 4.2. Influence of Endornavirus on the Ceratobasidium Small RNA Population

Up-regulated Cer-milRNAs may down-regulate target gene transcription. Gene targets of the significantly up-regulated Cer-milRNAs include transcription factors, suggesting an effect of viral infection on the control of regulatory networks and cellular growth and development of the fungus. Interestingly, we also found that protein kinase, Leucin-rich repeat (LRR) containing proteins are targets of up-regulated Cer-milRNAs. In plants, LRR proteins are involved in immune responses [46], in particular, NBS (nucleotide-binding site)-LRR proteins in plants provide recognition of pathogen products of avirulence (AVR) genes [47]. 

F-box protein-coding genes were also one of Cer-milRNA targets. Many studies have demonstrated that F-box proteins are important for fungal pathogenicity [48]. F-box proteins also contain LRR at their C-terminal protein-binding domains, responsible for interacting with the substrates. F-box proteins are found in quite a few pathogenic fungi. In the hemibiotrophic fungus Magnaporthe oryzae, the F-box protein is crucial for conidiogenesis, fungal growth and development, and for virulence [49]. Suppression of these proteins could result in suppression of the growth, development, and pathogenicity of the fungal host. Our previous findings show that the infection of three endornaviruses suppressed the growth of Ceratobasisidum in the laboratory environment [16].

Few transposable elements (TEs) were targets of 24 nt Cer-milRNAs-1 and 21 nt Cer-milRNA-3. Given the potential damage to genome stability caused by transposon movement, many organisms have developed defense systems that suppress TEs activity. Silencing of TEs has been found in plants, animals, and filamentous fungi. Especially in plants, transcriptional gene silencing (TGS) of transposons is mediated by 24-nucleotide heterochromatic (het)siRNAs, RDR2, DCL3 and AGO4 [50]. Thousands of transposon transcripts are specifically targeted by more than 50 miRNAs for cleavage and processing by RDR6 in Arabidopsis [51]. TEs are important to increase the adaptive ability of the fungi to the environment, coping with abiotic stresses (temperature, irradiation, and oxidative stress) or biotic, such as pathogen infection [52]. Effector genes of the fungi contain a high proportion of TEs that facilitate adaptation to the host or to new hosts [52,53,54]. Thus, it could be predicted that when Cer-milRNAs, which target a large number of TEs, were up-regulated, TE activity would be suppressed. This could affect the ability of the fungal coping with stress, including viral infections.

Although many mycoviruses are described having ‘latent’ or ‘mild’ effects on their hosts, there have been few studies investigating how such viruses affect gene expression of their hosts. In our attempt to do so in this fungal islate co-infected with three endornaviruses, we showed, not unexpectedly, that gene expression of the host is indeed influenced by the presence of apparently latent viruses. The great challenge now is to interpret how these influences at the translational level feed into an ecological system where a soil fungus interacts with an orchid plant as well as other soil organisms. There are enormous numbers of variables in any such systems, and our investigation into a small part of such a system must be placed in the context of natural selection of both the viruses and fungal host. Does the presence of the viruses provide a selective advantage to the host under at least some circumstances? Clearly, there remain many opportunities and challenges as we move towards an understanding of the roles of mycoviruses in natural systems. 

## Figures and Tables

**Table 1:** miRNA categories assigned *Ceratobasidium* small RNA loci after analysis by ShortStack.

Category	miRNA Prediction
N11	Possible mature miRNA had >5 unpaired bases in predicted precursor secondary structure.
N12	Possible mature miRNA was not contained in a single predicted hairpin
N13	Possible miRNA/miRNA* duplexes had >2 mismatches and/or >3 mismatched nucleotides
N14	Imprecise processing: Reads for possible miRNA, miRNA*, and their 3p variants added up to less than 50% of the total reads at the locus.
N15	Maybe. Passed all tests except that the miRNA* was not sequenced. Insufficient evidence to support a de novo annotation of a new miRNA family.
N5	Locus size is less than maximum allowed for RNA folding per option --foldsize (default is 300 nucleotides).
N6	Locus is not stranded (>20% and <80% of reads aligned to top strand)
N8	Strand of possible mature miRNA is opposite to that of the locus

miRNA* refers to the reverse strand.

**Table 2:** Features of the Ceratobasidium C02 genome.

Sequence and Assembly	
N50 (bp)	11,539
Maximum (bp)	190,502
Average (bp)	3980
Number of contigs	23,453
Number of contigs > 1000 bp with > 100× coverage	13,085
Genome size (bp)	93,339,198
GC content (%)	50
Number of predicted genes	31,294
Average transcript length (bp)	1353
Average number of exons per gene	5.8
Number of exons	182,873
Average exon size (bp)	226
Number of introns	151,579
Average intron size (bp)	76
Number of predicted proteins	32,405
Number of matches to InterPro proteins	27,593

**Table 3:** Summary of total small RNA sequencing data and reads mapped to the *Ceratobasidium* isolate C02 genome assembly.

Fungal Culture	Raw Reads	After Quality Control and Size Selection ^a^	Structural RNAs ^b^	Clean Mappable Reads ^c^	% strRNA	% Clean Mappable Reads	Count Mapped Reads ^d^	% Mapped Reads	Count Unmapped Reads	% Unmapped Reads
BCD2	12,280,779	6354430	1210857	5143573	19.06	80.94	4387783	85.31	755790	14.69
BCD7	12,018,693	5979763	1538344	4441419	25.73	74.27	3839643	86.45	601776	13.55
BCD8	16,243,640	7241215	2201059	5040156	30.40	69.6	4423800	87.77	616356	12.23
FREE5	13,576,160	6657187	1234775	5422412	18.55	81.45	5134752	94.69	287660	5.31
FREE6	14,654,508	7783981	1654271	6129710	21.25	78.75	5801256	94.64	328454	5.36
FREE8	15,329,619	7720742	1532205	6188537	19.85	80.15	5875387	94.94	313150	5.06

^a^ Number of reads that passed quality control steps, including adapter and base quality trimming, and were 18—30 nt long, ^b^ Number of reads mapped to Rfam databases, including tRNA, rRNA, snoRNA, snRNA, siRNA, etc. ^c^ Number of reads remaining after quality and size selection and removal of structural RNAs, ^d^ Number of raw reads mapped to the *Ceratobasidium* C02 genome.

**Table 4:** Differentially expressed milRNAs candidates predicted by ShortStack.

Codes	miRNA Prediction	Number of Small RNAs	Up-Regulated Small RNAs	Down-Regulated Small RNAs
N11	Possible mature miRNAs with >5 unpaired bases in a predicted precursor secondary structure.	154	64	0
N12	Possible mature miRNAs not contained in a single predicted hairpin	18	12	0
N13	Possible miRNA/miRNA* duplex with >2 bulges and/or >3 bulged nucleotides	8	5	0
N14	Imprecise processing: Reads for possible miRNA, miRNA*, and their 3p variants adding up to less than 50% of the total reads at the locus.	10	8	0
N15	Maybe. Passed all tests except that the miRNA* was not sequenced. Insufficient evidence to support a de novo annotation of a new miRNA family.	7	2	0

miRNA* refers to the reverse strand.

**Table 5:** *Ceratobasidium* C02 milRNA candidates identified by high-throughput sequencing.

milRNA Name	Sequence (5’-3’)	Length	milRNAs Location						miRNA	log2FC	padj	Diffexpressed
			Contig	Start	End	Length of Loci	Strand	Complexity				
Cer-milR-1a	GCACUUGUAGGCACCAAGCCUGUU	24	87	13,207	13,380	174	−	0.184	N14	0.928	8.20 × 10^3^	UP
Cer-milR-1b			88	9916	10,089	174	−	0.150	N14	0.802	3.33 × 10^2^	UP
Cer-milR-2a	UGGAGAUUACUUCAAGCGAA	20	2683	14,613	14,796	184	+	0.005	N14	1.666	3.91 × 10^10^	UP
Cer-milR-2b			3215	3411	3515	105	−	0.005	N14	1.716	1.31 × 10^11^	UP
Cer-milR-3	AGGUGCUCCCAGGCGCUUACGA	21	3197	5665	5849	185	+	0.303	N14	0.714	3.71 × 10^2^	UP
Cer-milR-4a	CCCAAAUUCACAUCCUGACA	20	3301	36,270	36,384	115	−	0.002	N14	2.012	2.36 × 10^26^	UP
Cer-milR-4b			4072	21,370	21,458	89	−	0.001	N15	2.000	4.95 × 10^25^	UP
Cer-milR-5	UCCCGGAGCACACGCUGGC	19	3301	38,139	38,234	96	−	0.171	N14	1.937	5.21 × 10^14^	UP
Cer-milR-6	UCCCGGAGCACGCGCUGGC	19	4072	23,216	23,286	71	−	0.065	N14	1.550	2.43 × 10^9^	UP
Cer-milR-7	UGUCAGUAGGAACAAUUG	18	5923	11,559	11,668	110	−	0.199	N15	0.659	4.76 × 10^2^	UP
Cer-milR-8	UGGUGACGACUGUGGGAUU	19	2085	8992	9046	55	−	0.007	N15	0.272	5.10 × 10^1^	NO
Cer-milR-9	ACUCUGUCAAGGCGAACA	18	2882	14,558	14,672	115	−	0.033	N15	0.420	3.69 × 10^1^	NO
Cer-milR-10	AUAUCCCGACUCAGGAGCUGGUCCG	25	3301	36,942	37,045	104	−	0.008	N14	−0.278	4.84 × 10^1^	NO
Cer-milR-11	AAUUUGAGCUUCCGGUCGAGCA	22	3879	1	204	204	−	0.217	N14	0.480	1.52 × 10^1^	NO
Cer-milR-12	UACGAGUCAAGAUGGUCAAGUUA	23	7096	3700	3981	282	+	0.008	N15	0.230	5.45 × 10^1^	NO
Cer-milR-13	UGUCUUCUGCAGUGGCCA	18	11,727	7908	8131	224	−	0.046	N15	0.161	7.61 × 10^1^	NO
Cer-milR-14	GGGCCAAAGUGCUUCGUA	18	18,920	1122	1290	169	−	0.048	N15	0.252	5.98 × 10^1^	NO

## Data Availability

The data is publicly-available under BioProject PRJNA873516 at NCBI.

## Supplementary Materials

The following supporting information can be downloaded at: https://www.mdpi.com/article/10.3390/v14102276/s1, Table S1: List of virus-specific primers used to identify Ceratobasidium endornavirus B (CbEVB), Ceratobasidium endornavirus C (CbEVC) and Ceratobasidium endornavirus D (CbEVD) in *Ceratobasidium* sp. C02 lines [1]. Table S2: Ten Ascomycotous fungal genomes with high-quality genome annotations were chosen to perform annotation of the *Ceratobasidium* C02 genome assembly. Table S3: Differentially expressed small RNA loci in *Ceratobasidium* C02 when the fungus is infected by Ceratobasidium endornavirus B, Ceratobasidium endornavirus C and Ceratobasidium endornavirus D. Table S4: Predicted target of statistically up-regulated Cer-milRNA of *Ceratobasidium* C02 when infected by Ceratobasidium endornavirus B, Ceratobasidium endornavirus C and Ceratobasidium endornavirus D. Table S5: Predicted targets by psRNATarget online tool of up-regulated Cer-milRNAs when compare virus-infected Ceratobasidium C02 cultures over the virus-free ones.
