# Supplementary material for: Small RNA Analyses of a Ceratobasidium Isolate Infected with Three Endornaviruses
Source: Viruses. 2022 Oct 17;14(10):2276. doi: 10.3390/v14102276 (PMC9610886; doi:10.3390/v14102276)
Supplement: Supplementary file 1 [file viruses-14-02276-s001.zip › Supplementary table S1 11 Oct SW.pdf]

Table S1. List of virus-specific primers used to identify Ceratobasidium endornavirus B (CbEVB), Ceratobasidium endornavirus C (CbEVC) and Ceratobasidium endornavirus D (CbEVD) in *Ceratobasidium* sp. lines. Primers were designed to amplify a region in the RNA-dependent RNA polymerase (RdRp) gene (Ong, Li et al. 2016).

Mycovirus[a]	Primer name	Primer sequence (5′ – 3′)	Expected amplicon length
CbEVB	EVB-RdRP-F	CCAATGCGTAGCAGACCTGT	918bp
	EVB-RdRP-R	GTTTCCGCACTTGATTVVAT	
CbEVC	EVC-RdRP-F	GCAATGAGGAGGCAGAAGAG	809bp
	EVC-RdRP-R	GCCAACATGCGAAGGAATAC	
CbEVD	EVD-RdRP-F	AGATTTGCAGAAGCGTGGTT	631bp
	EVD-RdRP-R	AGCGTGGATGATAAGGTTGG	

[a] CbEVB, Ceratobasidium endornavirus B; CbEVC, Ceratobasidium endornavirus C and CbEVD, Ceratobasidium endornavirus D
