# Supplementary material for: Small RNA Analyses of a Ceratobasidium Isolate Infected with Three Endornaviruses
Source: Viruses. 2022 Oct 17;14(10):2276. doi: 10.3390/v14102276 (PMC9610886; doi:10.3390/v14102276)
Supplement: Supplementary file 1 [file viruses-14-02276-s001.zip › Supplementary table S2 11 Oct SW.pdf]

Table S2. Ten Ascomycota fungal genomes with high-quality genome annotations were chosen to annotate the *Ceratobasidium* sp. C02 assembly

Genome	Accession	Reference	Source	Evidence
Laccaria bicolor	Lacbi2 (16097)	https://doi.org/10.1038/nature06556	JGI	*ab initio*<br>38,913 ESTs<br>protein homology - UniProtKB fungi
Mycena indigotica	GCF_014461135,1	https://doi.org/10.1073/pnas.2010761117	NCBI	*ab initio*<br>RNA sequencing<br>protein homology -UniProtKB fungi
Coprinopsis cinerea	Copci1	https://doi.org/10.1073/pnas.1003391107	JGI	*ab initio*<br>5,612 ESTs
Schizophyllum commune	Schco3	https://doi.org/10.1038/nbt.1643	JGI	*ab initio*<br>31,000 ESTs
Sparassis crispa	GCF_003851025.1	https://doi.org/10.1038/s41598-018-34415-6	NCBI	*ab initio*<br>RNA sequencing
Serpula lacrymans	SerlaS7_9_2	https://doi.org/10.1126/science.1205411	JGI	*ab initio*<br>undisclosed ESTs<br>protein homology - GenBank nr proteins
Postia placenta	Pospl1	https://doi.org/10.1073/pnas.0809575106	JGI	*ab initio*<br>44,520 ESTs<br>protein homology - 'reliable homology-based gene models'

Dichomitus squalens	Dicsqu463_1	https://doi.org/10.1128/MRA.00264-19	JGI	*ab initio*<br>RNA sequencing<br>protein homology - 'homology-based’
Agaricus bisporus var. *bisporus*	99190	https://doi.org/10.1073/pnas.1206847109	JGI	*ab initio*<br>79,271 ESTs<br>protein homology - GenBank nr proteins
