# Supplementary material for: Small RNA Analyses of a Ceratobasidium Isolate Infected with Three Endornaviruses
Source: Viruses. 2022 Oct 17;14(10):2276. doi: 10.3390/v14102276 (PMC9610886; doi:10.3390/v14102276)
Supplement: Supplementary file 1 [file viruses-14-02276-s001.zip › Supplementary table S5.pdf]

Table S5. Gene ontology terms analysis of the predicted gene targets of up-regulated Cer-milRNAs identified in *Ceratobasidium* sp. when infected with three endornaviruses: Ceratobasidium endornavirus B, Ceratobasidium endornavirus C and Ceratobasidium endornavirus D

Function	Term ID	Name
Biological process	GO:0006355	"regulation of transcription
	GO:1902600	"proton transmembrane transport"
	GO:0032508	"DNA duplex unwinding"
	GO:0006468	"protein phosphorylation"
	GO:0005975	"carbohydrate metabolic process"
	GO:0006351	"transcription
	GO:0006281	"DNA repair"
	GO:0000723	"telomere maintenance"
Cellular component	GO:0033179	"proton-transporting V-type ATPase
Molecular function	GO:0000981	"DNA-binding transcription factor activity
	GO:0005515	"protein binding"
	GO:0015078	"proton transmembrane transporter activity"
	GO:0016837	"carbon-oxygen lyase activity
	GO:0003678	"DNA helicase activity"
	GO:0047834	"D-threo-aldose 1-dehydrogenase activity"
	GO:0004672	"protein kinase activity"
	GO:0046983	"protein dimerization activity"
	GO:0020037	"heme binding"
	GO:0005506	"iron ion binding"
	GO:0008270	"zinc ion binding"
	GO:0003677	"DNA binding"
	GO:0005524	"ATP binding"
	GO:0016705	"oxidoreductase activity
